# Low-cost, sub-micron resolution, wide-field computational microscopy using opensource hardware

**DOI:** 10.1038/s41598-019-43845-9

**Published:** 2019-05-15

**Authors:** Tomas Aidukas, Regina Eckert, Andrew R. Harvey, Laura Waller, Pavan C. Konda

**Affiliations:** 1 Imaging Concepts Group, School of Physics and Astronomy, University of Glasgow, Scotland, G12 8QQ UK; 2 Department of Electrical Engineering and Computer Sciences, University of California, Berkeley California, 94720 USA

**Keywords:** Microscopy, Imaging and sensing

## Abstract

The revolution in low-cost consumer photography and computation provides fertile opportunity for a disruptive reduction in the cost of biomedical imaging. Conventional approaches to low-cost microscopy are fundamentally restricted, however, to modest field of view (FOV) and/or resolution. We report a low-cost microscopy technique, implemented with a *Raspberry Pi* single-board computer and color camera combined with Fourier ptychography (FP), to computationally construct 25-megapixel images with sub-micron resolution. New image-construction techniques were developed to enable the use of the low-cost Bayer color sensor, to compensate for the highly aberrated re-used camera lens and to compensate for misalignments associated with the 3D-printed microscope structure. This high ratio of performance to cost is of particular interest to high-throughput microscopy applications, ranging from drug discovery and digital pathology to health screening in low-income countries. 3D models and assembly instructions of our microscope are made available for open source use.

## Introduction

Low-cost, high-performance portable microscopes are essential tools for disease diagnosis in remote and resource-limited communities^1^. A fundamental requirement is to combine wide field of view (FOV) with the high resolution necessary for imaging of sub-cellular features of biological samples. This underpins efficient inspection of extended, statistically-significant areas for screening of, for example, cancer, malaria, or sickle cell anemia^2^. In conventional imaging, the number of pixels in the detector array constitutes a hard limit on the space-bandwidth product (SBP – the number of pixels in a Nyquist-sampled image^3,4^) so that increased FOV can be achieved only at the expense of reduced spatial resolution. SBP can be increased using larger detector arrays coupled with higher-performance, wide-field aberration-corrected optics, or by mechanical scanning, but these approaches add complexity, cost and bulk^5,6^.

Several low-cost portable microscopes have been proposed^7–12^, but they all suffer from the problem of small SBP. Early progress towards low-cost microscopy has involved the use of a high-cost microscope objective lens coupled to a mobile-phone camera^7^ and such instruments tend to suffer from a higher system cost, vignetting, short working distance, small depth of field (DOF) and narrow FOV. Lower-cost implementations have been reported in which the microscope objective is replaced by a camera lens from a mobile phone^8^, or a ball lens^9^, but their resolving power is limited by the small numerical aperture (NA) and high aberrations. Of these implementations, the use of mobile-phone camera lenses as objectives places an upper limit on the SBP: for example a 4-μm spatial resolution across 9 mm^2^ FOV corresponding a SBP of 2.25-megapixels^8^. The 4-µm resolution is insufficient for observing sub-cellular features and while a higher NA can be obtained using ball lenses, providing a resolution around 1.5 μm, they suffer from small SBP^8,13^.

**Figure 1:**
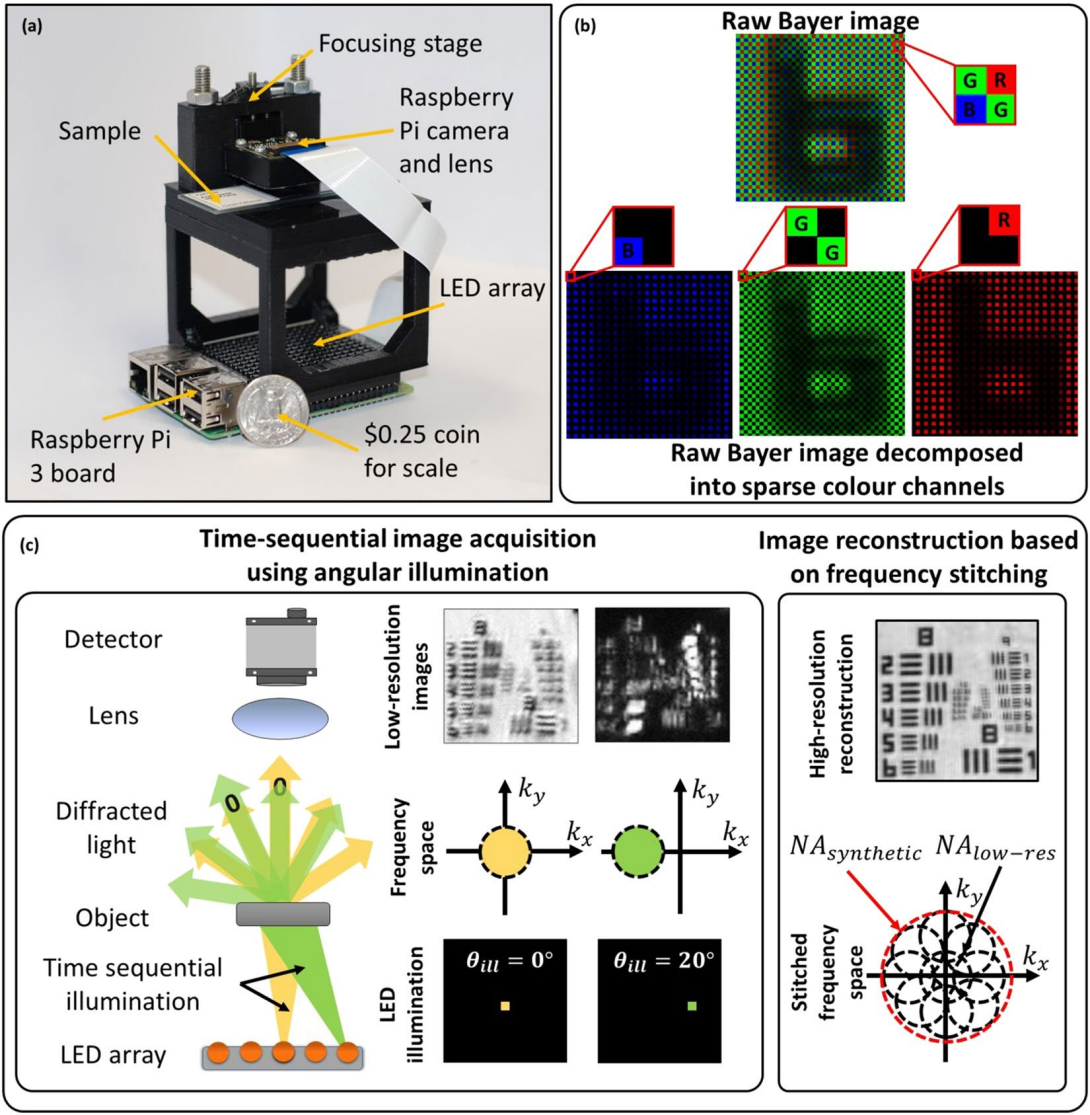
(**a**) Experimental setup next to a quarter US dollar for scale. Raspberry Pi 3 single-board computer board (placed at the bottom) enables wireless image acquisition and data transfer without the need for a PC. (**b**) Bayer color filter array indicating RGGB pixel arrangement. (**c**) In FPM several low-resolution images are obtained in time sequence, each illuminated with a corresponding to the object illuminated from a different angle. Angular diversity enables to obtain multiple frequency regions, which can be stitched together into a single high-resolution, wide-field image.

We report a low-cost, wide-field, high-resolution Fourier-ptychographic microscope (FPM)^14^, implemented with 3D-printed opto-mechanics and a *Raspberry Pi* single-board computer for data acquisition as shown in Fig. 1(a). High-SBP images are constructed from multiple low-resolution, detector-SBP limited images, captured in time-sequence using oblique illumination angles yielding a SBP that is much greater than that of the detector. We demonstrate 25-Megapixel microscopy using a 4-Megapixel detector array. The tilted illuminations provide translations of higher spatial-frequency bands into the passband of the objective lens^15^. Stitching of images in the frequency domain is implemented using an iterative phase-retrieval algorithm to recover high-resolution amplitude and phase of the sample image^16,17^, as well as aberrations due to the objective^14^. Recovery of phase information enables imaging of unstained transparent samples^18^ and computational calibration of illumination angles during image reconstruction is able to correct errors arising from misalignment of various components^19,20^, which is of particular importance for microscopy using low-cost 3D-printed devices.

In previous demonstrations of a low-cost 3D-printed FPM, the SBP was limited by the severe off-axis aberrations of the mobile-phone camera lens (1.5 µm resolution across 0.88 mm^2^ FOV giving a SBP of 1.56-megapixels), and employed a science-grade, high-cost monochrome sensor^21^. Exploiting the mass market for consumer color sensors in mobile phone cameras, we demonstrate the first use of a low-cost consumer color camera in FPM, to gain more than an order-of-magnitude cost reduction for an equivalent SBP. The main difference between the two sensor types is the spatial-spectral filtering provided by the Bayer filter array, which encodes recorded images into sparse red, green, and blue channels. While the decoding processes follows a standard demosaicing procedure (individual RGB channels are interpolated and stacked into a 3D matrix), the loss in image information due to sparse sampling requires special treatment within the FPM reconstruction algorithm. We address the sparse sampling problem and present new robust algorithms for calibrating the 3D printed system for high-quality image reconstruction. In addition, the *Raspberry Pi* single-board computer used for controlling the camera and illumination LEDs performs autonomous data acquisition, providing portability and compactness, such as is required for use inside incubation systems.

In the next section, simulations to study the impact of the Bayer filter array and the experimental results from our system are presented. Implications of the results and future directions are discussed in the later sections. The methods section includes descriptions of the experimental setup, data-acquisition, data processing and calibration procedures. We also include the necessary CAD files and an instruction set to build the FPM presented in this article (Supplementary Material S1).

## Results

The *Raspberry Pi* camera (a low-cost device that complements the *Raspberry Pi* computer) employs a low-cost CMOS sensor, such as is typically found in mobile phones. It employs a Bayer filter (red, green and blue filters arranged on a 2D matrix in a 2 × 2 RGGB pattern^22^ (Fig. 1(b))). This divides pixels on the sensor between the three color-filters resulting in sparsely sampled images: red channel −75% empty pixels, green channel −50% empty pixels and blue channel −75% empty pixels. The empty pixels are demosaiced (using bilinear interpolation) to produce a perceptually acceptable photographic image.

In FPM, the reconstruction algorithm^18^ (see Methods) involves a step to iteratively recover amplitude and phase of the low-resolution images, where the estimated amplitude is replaced by the experimentally obtained images. In color cameras, the experimental image has empty pixels (due to the Bayer filter) whose values are unknown. We have considered two approaches for mitigation of the sparse sampling due to the Bayer filter. The first, a *sparsely-sampled reconstruction* (SSR) algorithm^23^, updates only the non-empty image pixels, relying on the FPM reconstruction to estimate the empty image pixels. This approach increases the number of unknowns in the system and can have slower convergence or failure to converge. In a second approach, the empty pixels are estimated instead from demosaicing enabling the use of a conventional FPM recovery; we refer to this approach as *demosaiced reconstruction* (DR). With DR the interpolation errors introduced in demosaicing can introduce artefacts in the reconstruction. We report below a comparison of image-recovery accuracy using SSR and DR recovery applied to simulated data.

**Figure 2:**
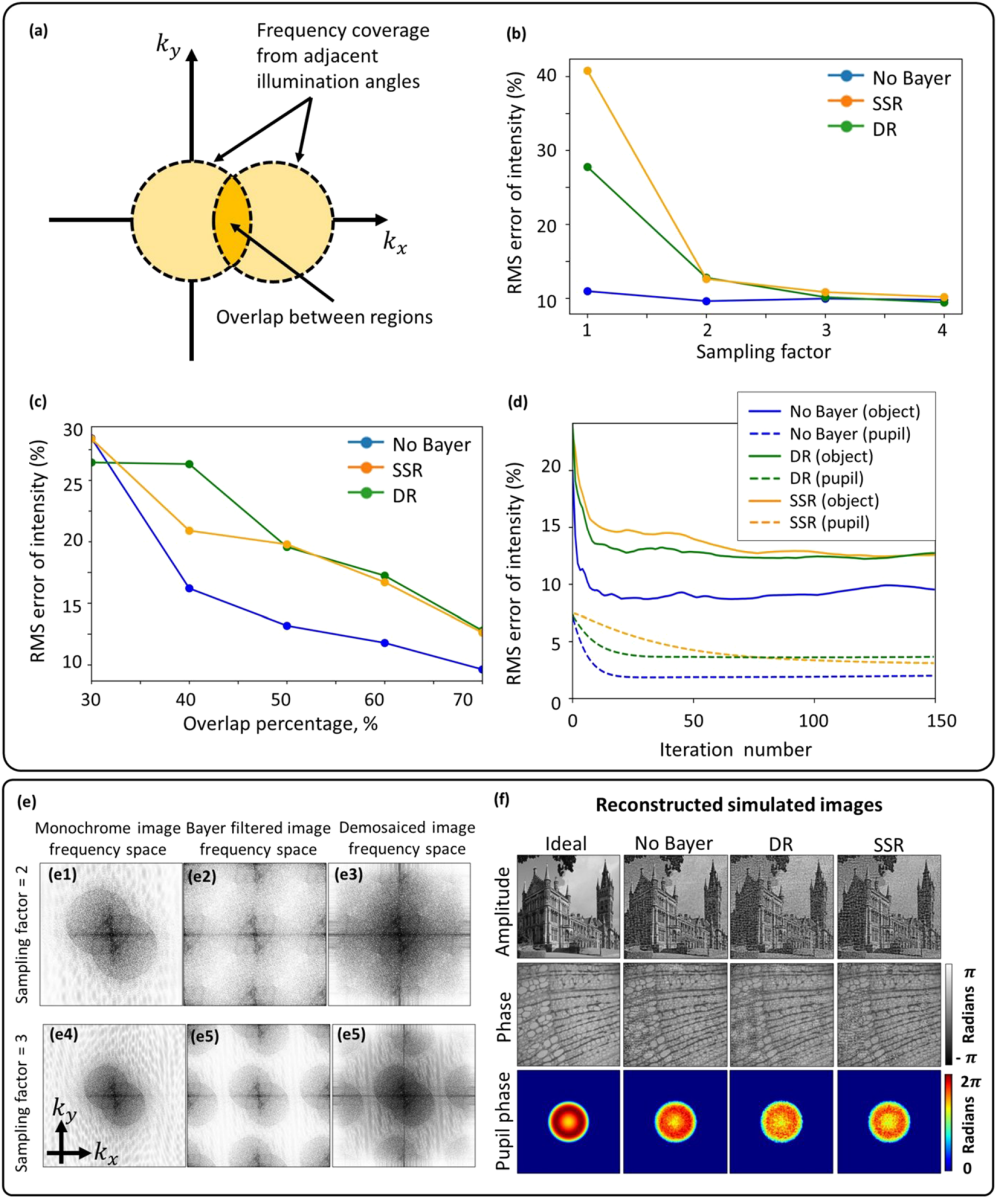
(**a**) Diagram illustrating what is meant by the overlap percentage between adjacent frequency regions. (**b**) Demosaiced and sparsely-sampled reconstruction accuracy for different sampling factors showing that a factor of two is required when using DR and SSR methods; 70% overlap area in the frequency domain. (**c**) Demosaiced and sparse reconstruction accuracy for different frequency overlap percentages. As expected, accuracy improves as overlap increases. (**d**) Reconstruction convergence plots for object amplitude and pupil phase (70% overlap and sampling factor of 2), indicating better performance of demosaiced reconstruction. (**e**) Frequency spectra of monochrome and color sensor images showing frequency replicates introduced by the Bayer filter and how it distorts the circular boundary. The boundary becomes undistorted only for a sampling factor of 3. (**f**) Reconstructed simulated images.

Convergence of the FPM reconstruction algorithms requires the experimental design conditions to satisfy Nyquist sampling of the image by the detector array and to have approximately 50% overlap between the frequency bands selected by adjacent illumination angles (Fig. 2(a))^24^. We assess here using simulations, how these requirements are modified by the reduced sampling rate associated with the sparse sampling of the Bayer matrix. Image quality is compared to recovery from non-Bayer-encoded images.

Using the far-field approximation^15^, the image intensity for a color channel can be written as

1 $$I(x,y)={|{ {\mathcal F} }^{-1}\{P({k}_{x},{k}_{y})\cdot  {\mathcal F} \{A(x,y){e}^{i\varphi ({\rm{x}},{\rm{y}})}\}\}|}^{2}\cdot B(x,y)+N(x,y)$$

where (*k*_*x*_, *k*_*y*_) are coordinates in frequency space, (*x*, *y*) are coordinates in real space, *P* is the pupil function, *A* and *ϕ* are the amplitude and phase distributions of the input object respectively, *B* is a binary mask corresponding to the color channel’s filter arrangement on the RGGB Bayer matrix, *N* is the added Gaussian image noise and F is the Fourier transform operator. Since robustness of the reconstruction is strongly dependent on the aberrations present in the pupil plane, they were simulated by including defocus and spherical optical aberrations generated using Zernike polynomials. We employed the Root-Mean-Squared (RMS) error between high-resolution reconstructed image and the expected ideal simulated image as a metric of image quality. We employed 150 iterations, which was more than sufficient for the FPM algorithms to converge.

In an imaging system, the image-sampling frequency is defined as *f*_*sampling*_ = *M*/*PS*, where *M* is the magnification and *PS* is the pixel size. This sampling frequency must satisfy the Nyquist sampling criterion, defined as twice the optical cut-off frequency, to avoid aliasing:

2 $${f}_{{sampling}}\ge {f}_{{Nyquist}}=2{f}_{{cut}-{off}}=2N{A}_{{obj}}/\lambda $$

The image-sampling frequency can be controlled in the experimental design by modifying the magnification since the pixel size is fixed by the camera sensor characteristics. To achieve the widest FOV possible without aliasing, the sampling factor (*f*_*sampling*_/*f*_*Nyquist*_) must be unity. For Bayer sensors, intuitively the effective pixel width is 2× larger due to the empty pixels in each color channel of the Bayer filter array, hence, the magnification needs to be increased by a factor of two compared to a monochrome detector array to compensate, i.e., the required sampling factor will be two. Since increasing the magnification reduces the FOV, simulations were performed (Fig. 2(b)) to assess whether the FPM reconstruction methods could converge with under-sampling to achieve the highest SBP.

### Comparison of FPM reconstruction techniques for bayer images

Sparsely-sampled reconstruction has been shown to be effective for aliased images with 75% sparsity^23^, offering an advantage in terms of maximum achievable SBP compared to demosaiced reconstruction. However, as can be seen in Fig. 2(b), the image reconstruction from Nyquist-sampled Bayer images exhibited large RMS errors of 20–30% compared to 10% for non-Bayer images. Reduced image quality for reconstruction from Bayer-sampled images is expected due to aliasing artefacts; however, these findings differ from the conclusions in^23^: probably due to practical differences in implementation, which did not involve compensation of optical aberrations and benefitted from low-noise data recorded by science-grade cameras. This enabled reconstruction of high-resolution images from data with 75% sparsity. However, in our implementation recovering the system aberrations and dealing with the detector read noise is crucial, hence, both SSR and DR reconstruction methods require an additional 2× magnification to satisfy the Nyquist sampling criterion.

In Fig. 2(c), the requirement for overlap between the spatial frequencies recorded by two adjacent LEDs is assessed. It suggests that RMS errors for SSR start to converge at ~40% overlap compared to 50% for DR; this is in agreement with the requirements for non-Bayer sensors^24^. Since the additional 2× magnification is used in these simulations, the frequency overlap requirement achieved is similar to the requirement for non-Bayer systems. Using these two optimal system parameters (2× additional magnification and a 70% frequency overlap), the overall convergence for DR and SSR and non-Bayer systems is compared in Fig. 2(d). It can be observed that DR has better convergence and pupil recovery than SSR. The RMS errors in the final reconstructions are close for DR and SSR, hence it can be concluded that DR has better convergence properties despite both reconstruction techniques resulting in similar reconstruction quality. All reconstructed images are shown in the Supplementary Material S2.

### Algorithmic self-calibration of LED misalignment

Our system is implemented using 3D printed components and intended to be portable; hence, it may become easily misaligned, affecting primarily the illumination angles (LED positions). In addition, image distortion and field curvature change the relative LED positions distinctly across the FOV^25^. We have implemented a recently-developed self-calibration algorithm for LED position misalignment correction^20^, solving the issues of image distortion and misaligned components with relatively good computational efficiency (see Methods). In this algorithm the intensity image of an off-axis illuminated brightfield image is Fourier transformed to produce two overlapping circles, centered around the illumination direction. Using image processing techniques, we can find centers of these circles providing a better calibration for the LED positions; hence, the calibration accuracy depends on how well these circles are delineated.

While a sampling factor of two is sufficient (for a monochrome sensor), our simulations suggest (Fig. 2(e)) that artefacts introduced by the Bayer matrix require the sampling factor to be around three to produce an undistorted circular boundary, regardless of demosaicing. The Bayer pattern can be treated as a periodic grating; hence, it produces frequency replicas (similar to diffraction orders), a type of aliasing artefact, which distort circle boundaries indicated by Fig. 2(e). Hence, by increasing the sampling frequency, the separation between these frequency replicas is increased to preserve the boundaries. In practice, the change in illumination wavelength varies the sampling factor for a fixed magnification since the sampling frequency is fixed but the Nyquist frequency changes; hence, 3× sampling factor requirement for red (630 nm) (enough for calibrating LED positions) results in 2× sampling factor in blue, the minimum required for overcoming Bayer sampling. This suggests that the red channel can be used for LED position calibration without losing additional SBP due to the increased sampling requirement. The FOV is divided into several segments and processed independently in FPM, hence the distortion is tackled by calculating the relative LED positions for each of these segments independently (see methods for the recovered distortion of the system).

## Experimental Results

Our FPM device (Fig. 1(a)) achieves high performance at low cost by use of mass-produced consumer electronics: a conventional mobile-phone-type color camera (with the lens displaced from the normal infinite-conjugate imaging position to enable short-range imaging), a *Raspberry Pi* single-board computer for data acquisition and an off-the-shelf LED array (*Pimoroni Unicorn Hat HD*) for synthesis of a programmable illumination that enables synthesis of a higher NA. The total component cost is about $150, but mass production of such a device would further reduce the component cost. The lens from the *Raspberry Pi Camera v2.0* provides 0.15NA and 1.5 × magnification when placed 7 mm from the object. A 16 × 16 array of LEDs with 3.3-mm pitch was located 60 mm below the object providing 0.4-NA illumination to enable synthesis of 0.55-NA FPM images. The FPM yields a 25-megapixel SBP: that is 870-nm resolution (*NA* = 0.55) - sufficient for sub-cellular imaging across a 4-mm^2^ FOV. FPM also enables multiple imaging modalities, including phase-contrast and darkfield imaging, combined with extended DOF and computational aberration correction^26,27^.

Computational correction of errors due to imperfect calibration (such as component misalignment and aberrations) is highly dependent on image quality, which is compromised by the Bayer matrix due to optical attenuation and spectral overlap and spectral leakage between the RGB channels. While signal-to-noise ratio was maximized by independent optimization of integration times for each illumination angle, the spectral overlap of the Bayer spectral filters was mitigated by each red, green and blue LED in a time sequence rather than simultaneously.

**Figure 3:**
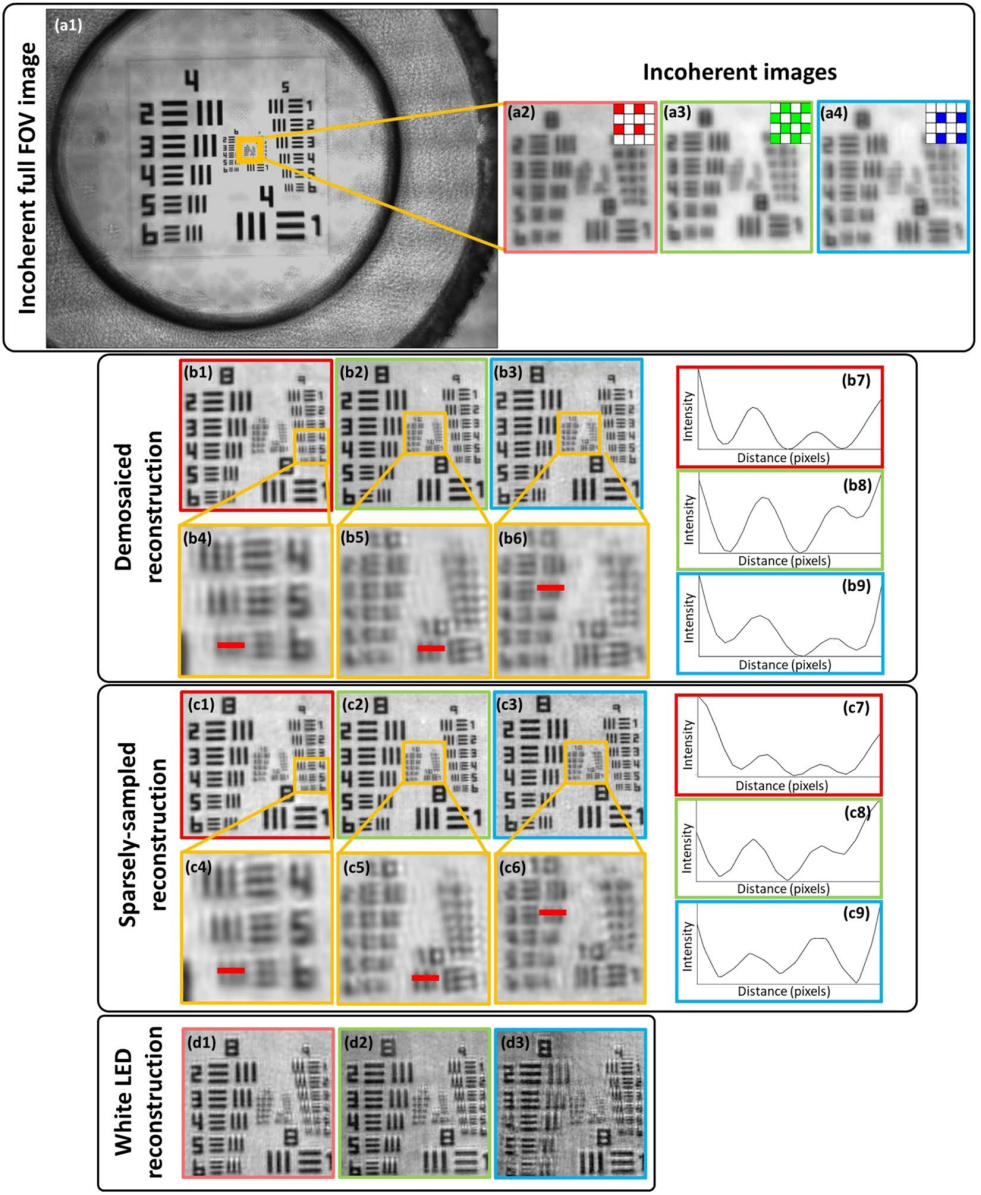
Reconstructions of a USAF resolution chart. (**a1**–**a4**) Incoherent raw images. (**b1**–**b9**) Demosaiced reconstructions and (**c1**–**c9**) sparsely-sampled reconstructions together with line profiles of the smallest resolved USAF target bars. The maximum achieved resolution using the blue LED was 780 nm based on group 10 element 3. (**d1**–**d3**) Reconstructed images with RGB LEDs used in parallel for illumination demonstrating the reduced reconstruction quality due to the spectral overlap between the color channels. The respective color channels are indicated by the red, green and blue borders of the left, middle and right images.

We used a standard USAF resolution test chart (Fig. 3(a)) to quantitively assess the performance and resolution improvement. Analysis of the reconstructed images shows a resolution improvement from group 8 element 4 (Fig. 3(a3)) to group 10 element 3 (Fig. 3(b6)) (using 470-nm (blue LED) illumination), which corresponds to a three-fold resolution improvement from 2.8 μm (incoherent-sum) to 780 nm. This resolution improvement is the result of the large synthetic NA offered by FPM, which is defined as *NA*_*FPM*_ = *NA*_*ill*_ + *NA*_*objective*_. Experimental results agree with the theoretical predictions, which give an increase in NA from *NA*_*coherent*_ = 0.15 (*NA*_*ill*_ = 0, *NA*_*objective*_ = 0.15) to *NA*_*FPM*_ = 0.55 (*NA*_*ill*_ = 0.4, *NA*_*objective*_ = 0.15). While reconstruction quality shown in Fig. 3(c1–c9, b1–b9) is nearly identical for both the DR and SSR, the DR offers faster convergence, since the SSR needs to iteratively recover the missing pixels that are readily available through demosaicing in DR. The impact of spectral overlap was demonstrated by illuminating the sample using RGB LEDs simultaneously (white light) and reconstructing each color channel. Artefactual reconstructions (Fig. 3(d1–d3)) are a result of the broken assumption of monochromatic light that is implicit in FPM and could be mitigated by a spectral multiplexing algorithm^28^.

**Figure 4:**
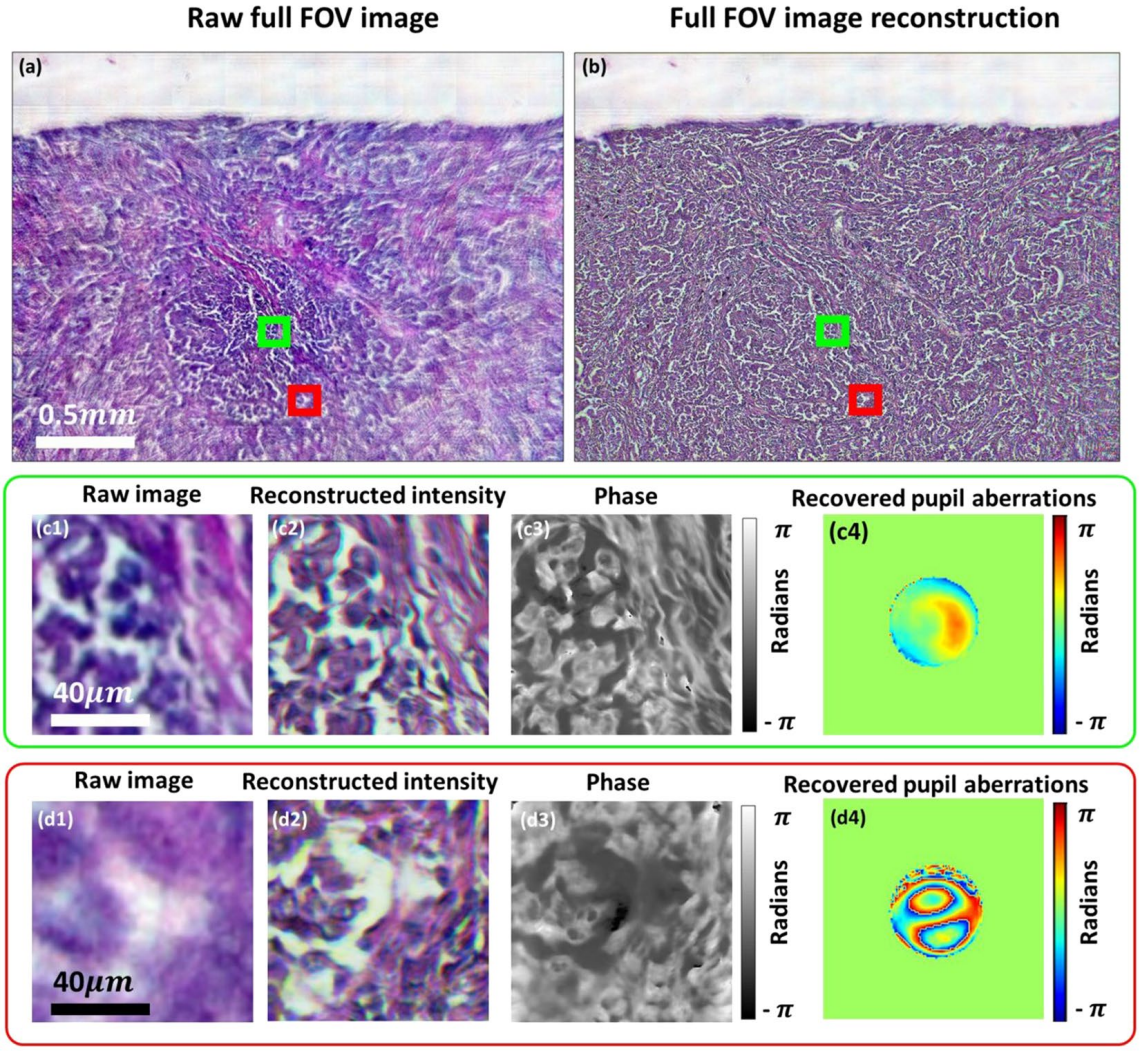
(**a**) Reconstructed and (**b**) raw lung carcinoma images. (**c1**,**d1**) are the captured raw, low-resolution images and (**c2**,**c3**,**d2**,**d3**) intensity and phase reconstructions for two different segments of the FOV. (**c4**,**d4**) Recovered pupils with aberrations.

Lastly, we have demonstrated experimentally that our reconstruction algorithms can compensate for high-levels of optical aberrations associated with the simple low-cost objective lens. Reconstructed images of a lung carcinoma (Fig. 4(a,b)) show high-quality reconstruction across the full FOV despite the presence of off-axis aberrations, which are recovered and corrected within the reconstruction procedure without requiring additional data. It can be observed clearly in Fig. 4(d1), that the raw image is severely aberrated compared to (c1), but the reconstruction (d2) is of similar quality to the central FOV section (c2). The phase images shown in Fig. 4(c3,d3) demonstrate the capability of imaging unstained samples. It can be seen from Fig. 4(a) that without aberration correction the FOV is limited by aberrations to a central area of ~1 mm^2^ while the FPM correction of imaging aberrations increases the usable area of the FOV by a factor of four.

## Discussion and Conclusion

We have described a demonstration of low-cost FPM, enabled by implementation using consumer-grade color cameras. We achieved a 4-mm^2^ FOV and 780-nm resolution (*NA* = 0.55), giving 25-megapixels SBP recovered from 256, 8-megapixel images. Compared to previous reports of low-cost, mobile microscopes^8^ the resolution of our system is a factor of 5 times better with the added advantage of a 4-times longer working distance (due to the low-NA lenses). Compared to systems where mobile-phone cameras are equipped with expensive microscope objectives^11^ our microscope offers 100-fold larger FOV without sacrificing resolution. Compared to a previously demonstrated 3D-printed FPM^21^, we report an increase in the FOV area by a factor 5 and resolution by almost a factor of 2, while the use of a color sensor instead of a more specialist monochrome sensor reduced the cost by 1–2 orders of magnitude. The improved performance of our system is made possible by improved aberration correction and calibration strategies capable of coping with simple, low-cost components^23^. It should be noted that (1) due to the additional magnification required by the Bayer filtering, the effective SBP achieved from each 8-megapixel image is only 2-megapixels and (2) the 25-megapixel SBP corresponds to the number of pixels in the image, but each pixel in the reconstructed image contains both amplitude and phase information. Although the recording of 256 images may seem a high number, this degree of redundancy is typical and necessary with FPM^14^, but can be reduced by a factor of up to 10 by using illumination multiplexing^29^.

Our stand-alone microscope weighs only 200 grams and has external dimensions of 6 cm × 9 cm × 11 cm. Data acquisition is autonomous offering major cost-savings and is ideal for applications such as cell-culture studies or point-of-care-testing applications that require field-portable devices. The *Raspberry Pi 3* computer-board enables wireless image acquisition, data transfer, and has potential for on-board FPM-based image reconstruction. Since image reconstruction is currently a computationally-intensive process we transferred the data to an external PC for processing, but in practice it would be possible to transfer the data onto a server network to perform the computations. Data transfer can be done in a few minutes during image capture itself, because it is not a CPU intensive process. The image reconstruction time depends on the computer being used. In FPM the image is divided into small segments and each of them is processed individually. A powerful workstation could process each segment in parallel taking less than a minute. Also, the use of a trained neural network for image recovery has been shown to improve image reconstruction speed by up to 50 times^30^, which is particularly attractive for systems with lower computational power. However, neural network use for medical applications requires an investigation into the availability of training datasets, or data overfitting^31^.

Total data acquisition time is determined by the integration times required to achieve a satisfactory signal-to-noise ratio and also by the number of frames required to achieve the required resolution and total number of pixels. To produce reconstructed images with 780-nm, 850-nm or 950-nm resolution we record 256, 196 and 144 images respectively. Capturing images using exposure times optimized for highest signal-to-noise ratio (rather than speed of image acquisition) took approximately 45 minutes (for 256 images), 30 minutes (196 images) and 15 minutes (144 images). The non-linear relationship is due to the use of longer exposure times for less-intense darkfield images. If a single exposure time is used for all images, the total acquisition time for 256 images could be reduced from 45 to 7 minutes, but with some reduction in image quality. An increase in the intensity of darkfield images, and consequent improvement in image quality, could however be achieved by use of a dome-shaped array^32^, where orientation of all LEDs towards the sample improves illumination efficiency, or by use of more intense LEDs.

Several additional refinements provide good scope for further reduction of image acquisition time. In our implementation, the readout of the *Raspberry Pi* image takes six times longer than the image integration time. Removal of this latency would reduce total image acquisition time to a little more than a minute. In addition, two parallelisation techniques can each provide an order-of magnitude reduction in data acquisition time. LED multiplexing^18,29^ enables acquisition with simultaneous illumination by LEDs at different angles. By multiplexing 8 LEDs only 32 images need to be captured instead of 256. Secondly, multi-camera Fourier ptychography^33,34^ enables parallel and simultaneous acquisition over an extended object-space numerical aperture, reducing acquisition time in proportion to the number of cameras used. It is therefore possible to reduce data acquisition times to significantly below a minute using the single-camera FPM architecture described here. By additional use of multi-camera techniques, acquisition times as short as one second could be possible. This would enable imaging of fast biological processes or rapid sampling of large biological samples, such as, for blood screening.

While conventional high-resolution microscopy requires a high-stability microscope structure to prevent relative motion between the sample and objective during data acquisition, our use of Fourier ptychography enables calibration of significant image shifts during data capture. Shifts are corrected as equivalent errors in LED positions during image reconstruction and this has enabled the use of a low-cost low-stability structure. We have compensated typical image drifts of about one pixel during the 256-image acquisition, but we have demonstrated reconstruction of high-quality images for shifts as large as 10 pixels.

We have demonstrated that Fourier ptychography can be performed by using low-cost commercial-grade Bayer color sensors, off-the-shelf consumer components and 3D-printed parts. This is enabled by robust pre-processing and reconstruction strategies. Moreover, we used a *Raspberry Pi 3* single-board computer for image acquisition and image transfer. The result is a highly compact, stand-alone microscope, with a component cost of $150, that is capable of wide-FOV, high-resolution imaging. The proposed microscope is suitable for cell-culture studies (its compactness enables it to fit inside an incubating chamber) and point-of-care diagnostics. Due to the simplicity of our setup, it is suitable for use as a teaching tool for computational optics in undergraduate labs and in research labs for conducting further research in FPM.

## Methods

### Experimental setup

Instructions for construction of our microscope shown in Fig. 1(a) can be found in Supplementary Material S1. To minimize the cost of our microscope we used easily accessible off-the-shelf, low-cost components. We chose a finite-conjugate microscope design because it requires only a single lens. Sample and focusing stages were custom designed and 3D-printed using a *Ultimaker 2* + 3D printer. A *Raspberry Pi V2 NOIR* camera module was used (8 megapixels, 1.12um pixel size) which contains a 3-mm focal-length camera lens, which was remounted and displaced from the sensor to achieve ~1.5× magnification. Frequency overlap of ~70% was obtained by placing the *Unicorn HAT HD* 16 × 16 LED array (3.3 mm pitch) 60 mm below the sample stage. The RGB LED array has peak illumination wavelengths of 623 nm, 530 nm, and 470 nm. The low-resolution microscope has 0.15 NA (providing 5-µm resolution at 470 nm), 2.42 × 1.64-mm^2^ FOV, and a 7-mm working distance. The synthetic NA achieved after FPM reconstruction was 0.55. Since the lens is used away from the intended infinite-conjugate position, the aberrations become progressively more severe toward the edges of the FOV. This could be mitigated be use of two back-to-back, co-aligned lenses^8^ with the penalty of reduced working distance and added experimental complexity.

### Data acquisition

Experimental low-resolution images were obtained using all 256 LEDs in the LED array. The *Python 3.6* programming language was used for the image acquisition via *picamera* package^35^, which enables the capture of raw 10-bit Bayer images^36^. Adaptive integration times for individual LEDs (longer for the off-axis LEDs towards the edges of the array) enabled enhancement of the dynamic range and image signal-to-noise ratio. We chose to transfer all 256 images obtained by the microscope from the *Raspberry Pi 3* computer onto a desktop *Windows* computer to speed up the reconstruction. Reconstruction could also be performed on the *Raspberry Pi* with necessary optimization of recovery algorithms.

### Image reconstruction

Recorded images were demosaiced using bilinear interpolation from the *OpenCV* processing package^37^ within the *Python 3.6* programming language. Before the reconstruction, the images were pre-processed by subtracting dark-frames to remove fixed pattern noise and all images were normalized according to their exposure times. The pre-processed images were divided into 128 × 128 pixel sub-images with an overlap of 28 pixels between adjacent image segments to aid in seamless stitching of the high-resolution reconstructions. Finally, LED-position calibration is performed independently on each image segment as described in the next section.

**Figure 5:**
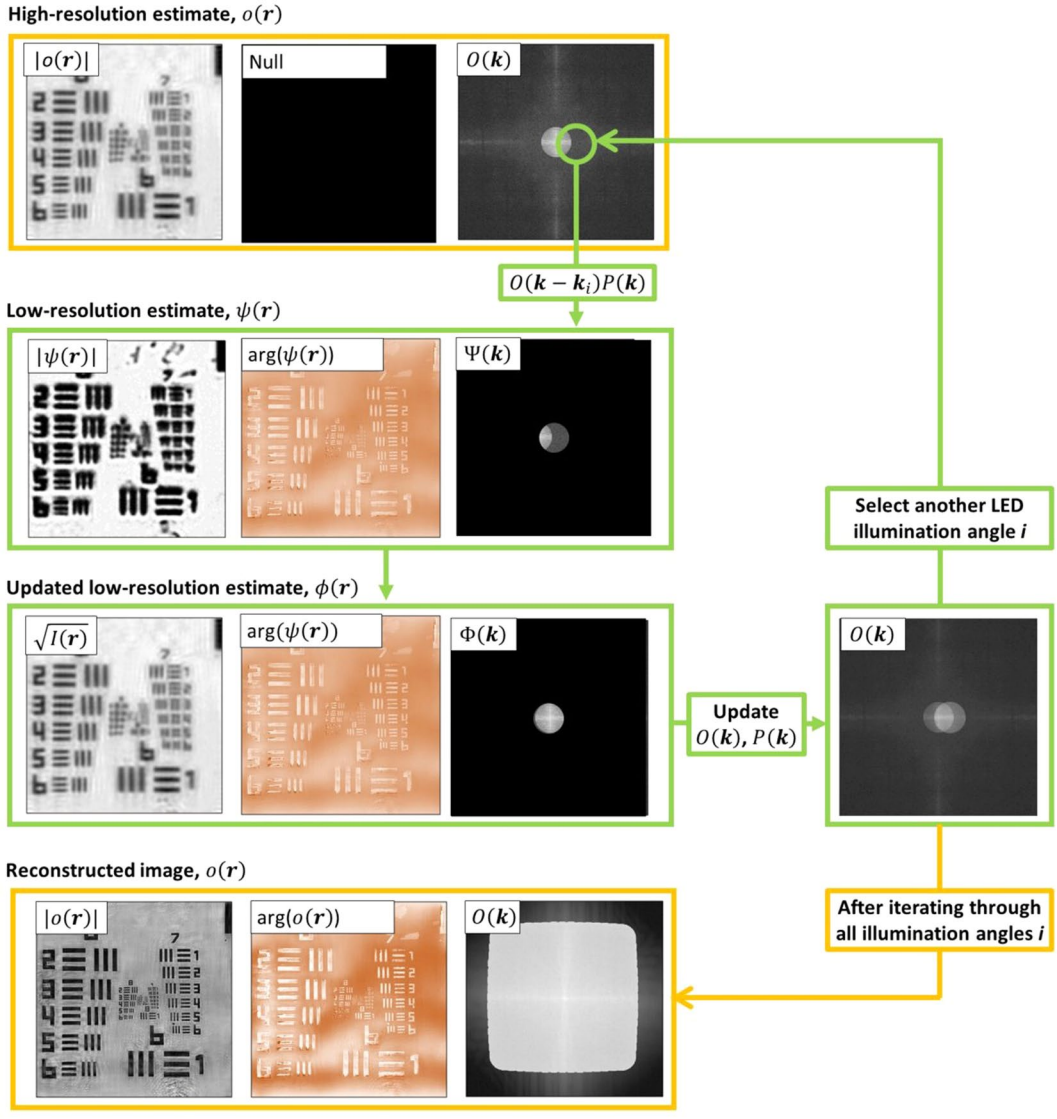
Diagram of a single FPM reconstruction algorithm iteration *n*. It starts by initializing a high-resolution image estimate ***o***_0_(***r***), which was an interpolated experimental brightfield image. For each illumination angle, a region of the high-resolution estimate spectrum, corresponding to illumination angle *i* is low-pass filtered to produce an estimate of the low-resolution image: ${{\rm{\psi }}}_{{\boldsymbol{n}}}^{({\boldsymbol{i}})}$. A new estimate, ${\varphi }_{{\boldsymbol{n}}}^{({\boldsymbol{i}})}$ is obtained by replacing the amplitude of $\,{{\rm{\psi }}}_{{\boldsymbol{n}}}^{({\boldsymbol{i}})}$ with the amplitude of the demosaiced recorded image, $\sqrt{{\boldsymbol{I}}}$, while retaining the phase of ${{\rm{\psi }}}_{{\boldsymbol{n}}}^{({\boldsymbol{i}})}$. The low-resolution spectrum is added into the high-resolution spectrum using eqs (6) and (7) to yield the updated object spectrum and pupil functions *O*_*n*+1_(***k***) and *P*_*n*+1_(***k***). The whole process continues till spectrum regions corresponding to all illumination angles are updated. An example of the reconstructed spectrum is shown at the bottom of the figure.

The FPM reconstruction algorithm is performed on each section of the FOV referred to as *I*^(*i*)^(***r***), where ***r*** is the coordinate vector in object space and *i* is the index corresponding to the LED used to illuminate and obtain the image. Before the reconstruction a high-resolution, wide-field object *o*(***r***) and its Fourier spectrum $O({\boldsymbol{k}})= {\mathcal F} \{o({\boldsymbol{r}})\}$ are initialized by interpolating one of the low-resolution images to the required dimensions, where ***k*** is the coordinate vector in k-space and ${\boldsymbol{ {\mathcal F} }}$ is the Fourier-transform operator. The reconstruction steps described below are repeated for multiple iterations and within an *n*^th^ iteration, images obtained from illumination angles *i* are stitched together using the following steps shown in Fig. 5:

Create a low-resolution target image Fourier spectrum estimate Ψ(***k***) by low-pass filtering the high-resolution, wide-field spectrum estimate with the pupil function *P*(***k***)

3 $${{\rm{\Psi }}}_{n}^{(i)}({\boldsymbol{k}})={O}_{n}({\boldsymbol{k}}-{{\boldsymbol{k}}}_{i}){P}_{n}({\boldsymbol{k}}),$$

where ***k***_*i*_ is the k-space vector corresponding to angular LED illumination with an index *i*. Therefore, ${{\rm{\Psi }}}_{n}^{(i)}({\boldsymbol{k}})$ is the *n*^th^ estimate of the recorded complex image spectrum at frequency ***k*** corresponding to the band of object frequencies centered on ***k***–***k***_*i*_.

Create a low-resolution target estimate ${{\rm{\psi }}}_{n}^{(i)}({\boldsymbol{r}})={ {\mathcal F} }^{-1}\{{\Psi }_{n}^{(i)}({\boldsymbol{k}})\}$ and use it to create the updated low-resolution estimate ${\varphi }_{n}^{(i)}({\boldsymbol{r}})$ by replacing its amplitude with the experimentally obtained one:

4 $${\varphi }_{n,\,SSR}^{(i)}({\boldsymbol{r}})=(\sqrt{{I}^{(i)}({\boldsymbol{r}})\cdot B({\boldsymbol{r}})}+{| \psi }_{n}^{(i)}({\boldsymbol{r}})\cdot (1-B({\boldsymbol{r}}))| )\frac{{\psi }_{n}^{(i)}({\boldsymbol{r}})}{|{\psi }_{n}^{(i)}({\boldsymbol{r}})|},$$

where B(**r**) is the binary Bayer matrix for the color channel being reconstructed. This is required if SSR is used^23^, otherwise, if DR is being used then setting *B*(***r***) = 1 results in the standard amplitude update step

5 $${\varphi }_{n,\,DR}^{(i)}({\boldsymbol{r}})=\sqrt{{I}^{(i)}({\boldsymbol{r}})}\frac{{\psi }_{n}^{(i)}({\boldsymbol{r}})}{|{\psi }_{n}^{(i)}({\boldsymbol{r}})|}.$$

Create an updated low-resolution Fourier spectrum

6 $${{\rm{\Phi }}}_{n}^{(i)}({\boldsymbol{k}})= {\mathcal F} \{{\varphi }_{n}^{(i)}({\boldsymbol{r}})\}.$$

Update the high-resolution object Fourier spectrum *O*(***k***) using a second-order quasi Newton algorithm^38^ together with embedded pupil recovery (EPRY)^16^ and adaptive step-size^39^ schemes to improve convergence

7 $${O}_{n+1}({\boldsymbol{k}})={O}_{n}({\boldsymbol{k}})+{\alpha }^{n}\frac{|{P}_{n}({\boldsymbol{k}}+{{\boldsymbol{k}}}_{i})|{P}_{n}^{\ast }{\boldsymbol{k}}+{{\boldsymbol{k}}}_{i}}{{|{P}_{n}({\boldsymbol{k}})|}_{{\max }}\,({|{P}_{n}{\boldsymbol{k}}+{{\boldsymbol{k}}}_{i}|}^{2}+{\delta }_{1})}{\rm{\Delta }},$$

8 $${P}_{n+1}({\boldsymbol{k}})={P}_{n}({\boldsymbol{k}})+{\beta }^{n}\frac{|{O}_{n}({\boldsymbol{k}}-{{\boldsymbol{k}}}_{i})|{O}_{n}^{\ast }({\boldsymbol{k}}-{{\boldsymbol{k}}}_{i})}{{|{O}_{n}({\boldsymbol{k}})|}_{max}\,({|{O}_{n}({\boldsymbol{k}}-{{\boldsymbol{k}}}_{i})|}^{2}+{\delta }_{2})}{\rm{\Delta }},$$

9 $${\rm{\Delta }}={{\rm{\Phi }}}_{n}^{(i)}({\boldsymbol{k}})-{{\rm{\Psi }}}_{n}^{(i)}({\boldsymbol{k}}),$$

where *δ*_1_, *δ*_2_ are regularization parameters and *α*, *β* are adaptive-step size constants which are selected to improve convergence. More details on the pupil-aberration recovery framework are given in the following sections.

All reconstructed sections were stitched together to produce a full-FOV reconstructed image. Alignment and contrast variations were corrected prior to stitching. Histogram equalization with the central section is performed to remove contrast variations across the FOV for both amplitude and phase. Finally, all sections are blended together using *ImageJ* (using the *Fiji* plugin package)^40^ to produce full-FOV images with seamless stitching.

All steps described above were performed for each of the red, green and blue channels independently and the final color image was assembled using linear image alignment with the scale-invariant feature transform (SIFT, part of the *Fiji* plugin package within *ImageJ*)^40^ for each channel and mapping them into RGB color panes.

### Computational calibration of LED positions

**Figure 6:**
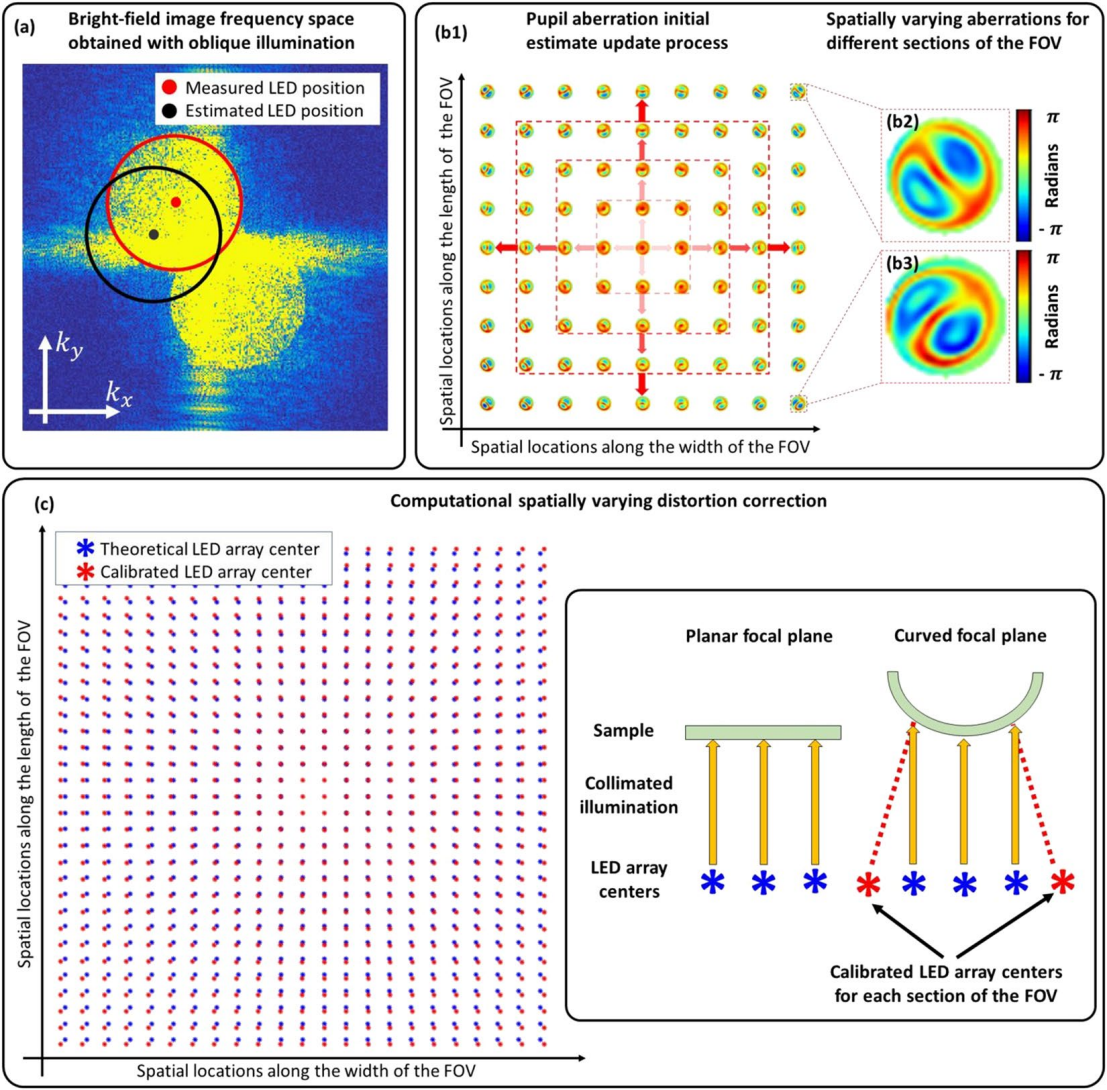
(**a**) Frequency space of a brightfield image obtained using an oblique illumination angle. Blue and green dots indicate initial and corrected LED positions respectively by using circle fitting. (**b1**) Aberrations recovered from each section are used as initial estimates for neighboring sections, starting from the center of the FOV towards the edges. (**b2**,**b3**) Examples of recovered aberrations throughout the full-FOV indicating spatially-varying aberrations. (**c**) Implementing LED calibration on each segment across the FOV enabled us to find the spatially varying distortion by measuring the global LED position shift.

An LED self-calibration method based on frequency-spectrum analysis of bright-field images^20^ was used to locate pupil positions in spatial-frequency space for every 128 × 128 pixel section of the image, in order to accurately estimate the angle of illumination at the sample associated with each LED. A microscope objective acts as a low-pass filter and off-axis illumination shifts the frequencies in the object plane corresponding to the frequencies transmitted by the objective, enabling recording of higher spatial frequencies. These higher frequencies within the brightfield region appear as two overlapping circles in the Fourier transform of the intensity image, centered at the spatial frequency of the illumination angle. Finding the center of these circles yields the LED positions with sub-pixel accuracy, for every brightfield illumination angle^20^ (Fig. 6(a)). After finding position displacements for each bright-field LED, a homographic transformation matrix that best represents the misalignment of the LED array is derived. This transformation matrix is applied to dark-field LEDs as well. However, non-linear distortions, such as field curvature^25^, make LED positions appear to be distorted differently across the FOV. To mitigate this problem, we split the full FOV image into 128 × 128 pixel sections and apply LED calibration for each section individually. If non-linear distortions are present, then each section will have a different LED array translation shown in Fig. 6(c). These distortions were corrected using an affine transformation that best represents corrections for each section of the FOV.

### Computational aberration correction

Spatially-varying aberrations for each segment of the FOV are recovered using the EPRY algorithm^16^ to enable FPM reconstruction of the images. However, our microscope suffers from aberrations that increase progressively towards the edges of the FOV, and the EPRY algorithm fails for the more highly aberrated sections. A good initial estimate of the aberrations is required for the EPRY algorithm to converge. Therefore, starting with the central 128 × 128 section of the FOV, we run the EPRY recovery step for 40 iterations, reset the recovered image intensity and phase while retaining the aberrations, and iterate the algorithm for 3 more times. The reset step forces the algorithm to escape from local minima and enables convergence towards a global solution. We use the recovered central aberrations as an initial estimate for the surrounding sections (Fig. 6(b)). This update process continues until aberrations for every section of the FOV are recovered.

Low-cost lenses, such as the ones we have used, tend to suffer from severe chromatic aberrations. We found that when the microscope is focused using one color of LED, the chromatic aberration (primarily defocus) for images recorded using other colors was significant to cause the reconstruction algorithms to fail. The aberrations recovered from the central section of the color where the microscope is focused are used as an initial estimate for the defocused color that is being processed. This involved decomposition of recovered pupil aberrations into 30 Zernike coefficients using the singular value decomposition function in *MATLAB* from which the chromatically-aberrated pupil functions were estimated.

## Supplementary information

Supplementary Material

## Data Availability

Data acquisition codes and 3D-printed designs can be obtained from 10.5525/gla.researchdata.594. Raw data and computer codes for EPSRC-funded research requirements were uploaded to 10.5525/gla.researchdata.687.
