# Supplementary material for: Low-cost, sub-micron resolution, wide-field computational microscopy using opensource hardware
Source: Sci Rep. 2019 May 15;9:7457. doi: 10.1038/s41598-019-43845-9 (PMC6520337; doi:10.1038/s41598-019-43845-9)
Supplement: Supplementary file 1 — Supplementary Material [file 41598_2019_43845_MOESM1_ESM.pdf]

Low-cost, sub-micron resolution, wide-field computational microscopy using opensource hardware

Tomas Aidukas[1], Regina Eckert[2], Andrew R. Harvey[1*], Laura Waller[2], Pavan C. Konda[1]

[1] Imaging Concepts Group, School of Physics and Astronomy, University of Glasgow, Scotland, G12 8QQ, UK
[2]Department of Electrical Engineering and Computer Sciences, University of California, Berkeley, California 94720, USA
**Andy.Harvey@glasgow.ac.uk*

Supplementary material 1: Instructions to build a *Raspberry Pi* Fourier ptychographic computational microscope

This document provides instructions to build a low-cost computational microscope reported in the manuscript: "*Low-cost, sub-micron resolution, wide-field computational microscopy using opensource hardware*". The CAD files and data acquisition codes can be downloaded from http://dx.doi.org/10.5525/gla.researchdata.594.

Introduction

One of the aims when building this microscope was to use only off-the-shelf components that can be easily bought anywhere and to design the microscope in such a way that it could be assembled with minimal external components. Avoiding complexity allowed us to build a very low-cost and robust microscope, which can be assembled and used with opensource software. Designs for the parts were made using *OpenSCAD* open source CAD software and printed with *Ultimaker 2+* 3D printer.

The microscope was designed around the *Raspberry Pi 3* computer board due to a wide opensource community and the support available. The computer itself has a CSI port to which a *Raspberry Pi* camera can be connected. For the illumination we used a *Unicorn HAT HD* 16x16 LED array, which is an add-on designed for the *Raspberry Pi* boards. It mounts directly onto the GPIO pins on top of the board. Camera and the LED board can be connected and controlled easily via opensource libraries available for *Python* or C++ programming languages.

Furthermore, *Raspberry Pi* camera comes mounted with a mobile-phone-camera type lens. It was unscrewed from the camera and used as our microscope objective. The component list required to build the setup is provided below, along with a step-by-step instruction set for assembly and operation of the microscope.

Component list

Off-the-shelf components	Quantity	Purpose	Suppliers and manufacturers
Raspberry Pi 3 computer board (~$32)	1	Controlling the camera and LED array; image capture and storage	Multiple suppliers (e.g. Pimoroni, Farnell, RS)
20mm long M3 screws (unit price of ~$0.05)	4	Fix Raspberry Pi computer board to a stable base	Multiple suppliers (e.g. RS)
Unicorn HAT HD 16x16 LED array (~$30)	1	Illumination source	Pimoroni
Raspberry Pi V2.1 NoIR camera (~$20)	1	Image sensor	Multiple suppliers (e.g. Pimoroni, PiHut)
Unscrewed lens from the Raspberry Pi camera (Part 4)	1	Microscope objective	
3.5mm diameter, >10mm long, 0.25mm pitch screw and bushing (Thorlabs F3ES25, F3ESN1P) (~$7, could use a regular screw to reduce cost)	1	Used for high-accuracy focusing	Thorlabs
>40mm long M6 screws + nuts (unit price of ~$0.05)	2	Attaching the focusing stage to the sample stage	Multiple suppliers (e.g. RS)
5.6mm diameter, ~10mm long springs (RS Stock No. 821-431) (~$1, or any other stiff spring)	2	Counter balance force for the focusing stage	Multiple suppliers (e.g. RS)
5mm long M1.5 screws (unit price of ~$0.05)	4	Screwing *Raspberry Pi* (part 4) camera to the focusing stage	Multiple suppliers (e.g. RS)

Supplementary Table S1 1. List of the off-the-shelf components required to build the microscope. Several links for the products are provided at the end of the document.

Design

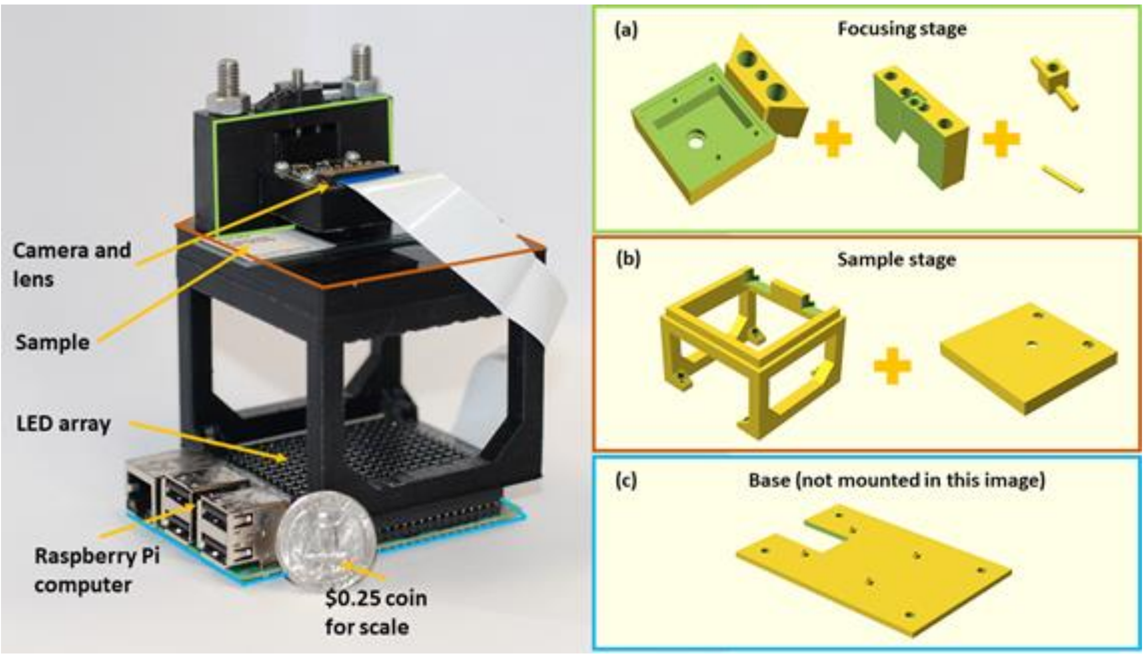

Supplementary Figure S1 1. The experimental setup with all the necessary annotations for reference to 3D printed designs.

Supplementary Figure S1 1 shows the assembled setup together with 3D printed parts required. It was built using components described in Supplementary Table S1 1. Once each component is 3D printed, the assembly is very simple and requires only a few screwdrivers.

Base

The plastic base shown in Supplementary Figure S1 1(c) was printed such that the Raspberry pi and the sample stage could be screwed onto it. The base itself has 4 holes which can be used for screwing the microscopes to the optical bench if needed. This was designed to provide higher stability when longitudinal imaging might be required.

Sample stage

The sample stage shown in Supplementary Figure S1 1(b) was designed to be mounted on the Raspberry Pi board with an LED array on top. The four screw holes on the 3D printed sample stage match those found on the Raspberry Pi and the plastic base. All components can be screwed tightly together to form a single microscope unit.

There is another 3D printed part that goes on top of the sample stage legs. It has 3 holes on it where the central one acts as an aperture for the sample, reducing any stray light and reflections from the LED array. The other 2 holes were made for screws that attach the focusing stage to the sample stage.

Camera holder and focusing stage

The focusing stage shown in Supplementary Figure S1 1(a) is composed of four 3D printed elements.

The camera holder module (the first 3D printed part seen in Supplementary Figure S1 1(a)) was designed to mount the microscope objective (the unscrewed camera lens) in place and screw the camera above it. This was designed for finite-conjugate microscope configuration to be established. The unscrewed lens from the camera has a 1.5mm aperture only on one side; lens must be mounted such that the aperture is facing downwards (towards the sample). This compact design was set to achieve 1.5× magnification, but it can be easily modified by changing the distance between the lens and the detector. It should be noted that the sensor is not glued to the camera board well. To ensure correct alignment it is best to re-glue the sensor.

The camera holder mount, (the second 3D printed part seen in Supplementary Figure S1 1(a)) serves several purposes including focusing the sample. Firstly, it has rails onto which camera holder module is mounted and can be moved up or down for focusing. The central hole in the camera holder mount is for the 0.25mm pitch screw. Springs are fed through the inner pair of holes in the camera holder mount and the corresponding holes in the camera holder module. They are held in place by sliding the pins shown in Supplementary Figure S1 1(a) through each end of both springs. The screw is used to push down on the camera holder module while the springs and bottom pin provide a counter force to push it upwards. This way the module can slide along the rails with high-precision, by turning the screw. Springs provide stability and push the module upwards when the screw does not provide a downward force anymore, which should minimize the backslash error.

Secondly, the outer holes in the camera holder mount enables addition of screws or bolts to attach the whole focusing module to the top of the sample stage. While the focusing is done via a translation stage, the sample must be translated by hand. In our setup, the FOV is large so precise translation is not required; hence, we chose to use this design. However, there are 3D printed sample translation stages available in the opensource community that can be integrated into our design.

Assembly instructions

Access to a 3D printer is required to print several parts required for the assembly. We used *Ultimaker 2+* with a nozzle size of 0.25mm for the camera holder module and 0.4mm for the other components. Also, the lens from the *Raspberry Pi V2.0* camera must be unscrewed before the assembly. Step-by-step instructions to assemble the microscope:

1. 3D print all the parts using a printer of your choice. We used *openSCAD* to design, render and save the designs in .STL format. *CURA* software was used to create the files that can be read by the *Ultimaker 2+* 3D printer. Black PLA filament and a 0.4mm diameter nozzle was used for printing the sample stage parts, while a 0.25mm nozzle was used to print the focusing stage. Our files were designed to match the tolerance of the nozzles on our printer. The 3D models need to be tweaked when a different nozzle size or a different 3D printer is used due to change in the tolerances.

2. Connect the *Raspberry Pi* camera to the *Raspberry Pi* board.

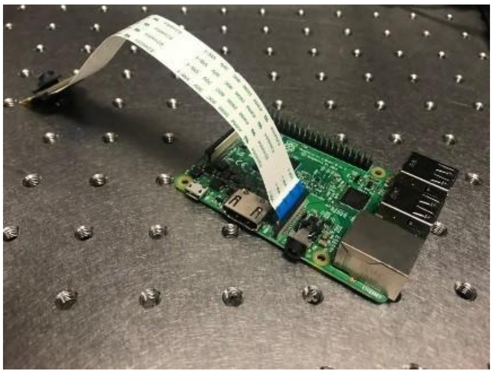

3. Mount the LED array on top of the Raspberry Pi board by plugging it into the GPIO pins on the board.

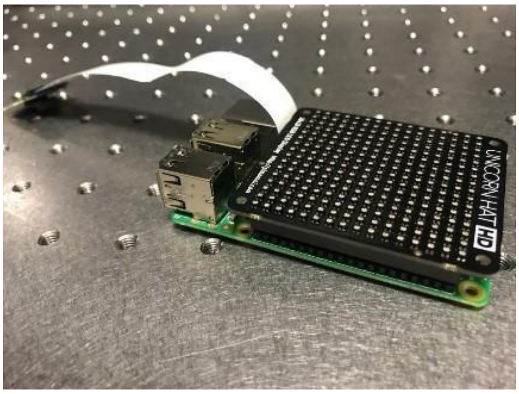

4. Place the sample stage such that the screw holes match the base; making sure that the sample-stage feet are not on top of any of the LEDs. Then place a nut in each foot of the sample stage.
5. Place the spacers in between the *Raspberry Pi* board *UnicornhatHD* board so that they are aligned with the screw holes and then screw the *Raspberry Pi* board and the sample stage to the base such that they form a single rigid module.

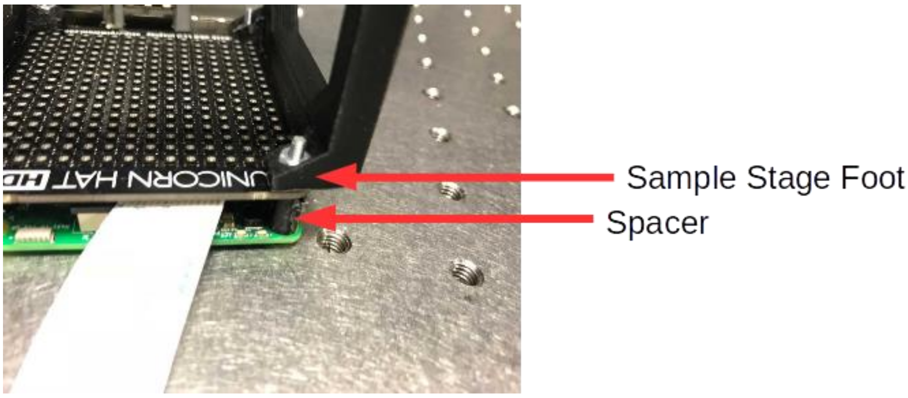

6. Take the camera holder; place the lens in the circular slot with the aperture facing downwards, towards the sample stage.

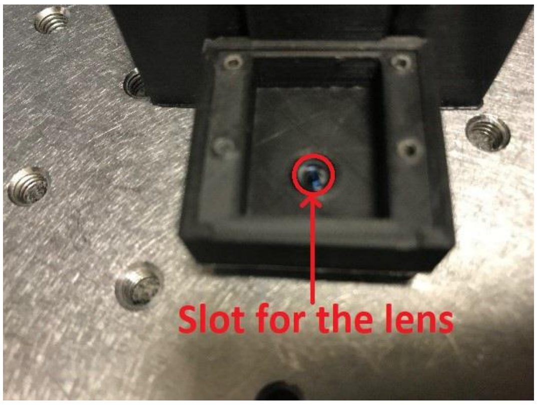

7. Mount the camera, align with the screw holes of the 3D printed camera holder; screw it in place tightly.

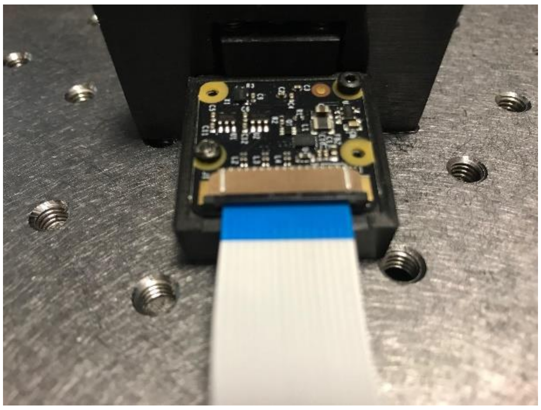

8. Slide the camera holder module onto the focusing stage rails. Thread the springs through the holes on the focusing stage and the camera module as shown by a red arrow in the figure below. Use two 3D printed horizontal pins seen in Supplementary Figure S1 1(a)) to hold the ends of the springs at the top and bottom of the focusing stage; the spring should be long enough such that it is stretched out and apply a strong counter-balance force to the screw.

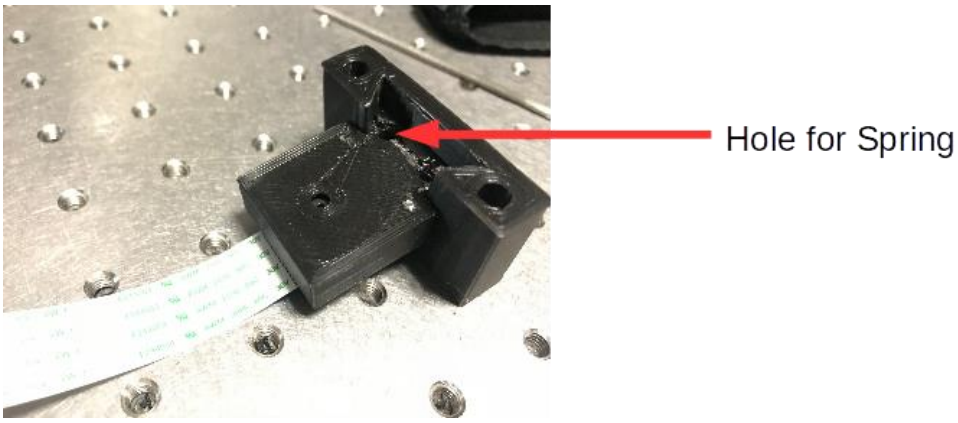

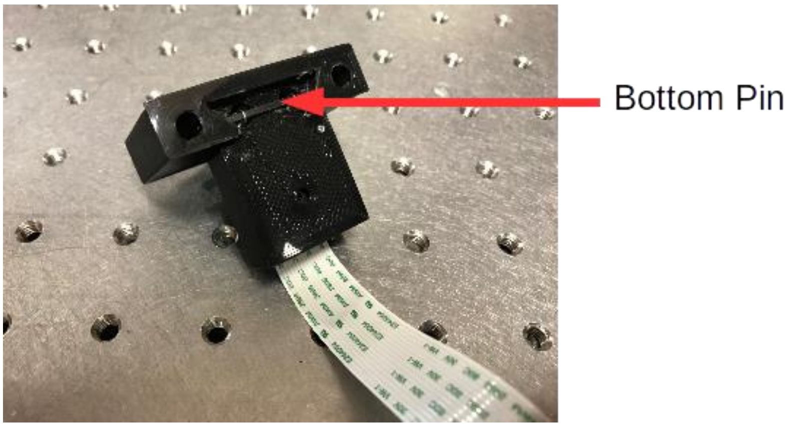

9. Pull down the camera holder module and, from underneath, place the bushing up into the central hole of the focusing stage. Then, place the screw in the top of the camera holder mount. Screw it in such that the screw pushes onto the camera holder module.

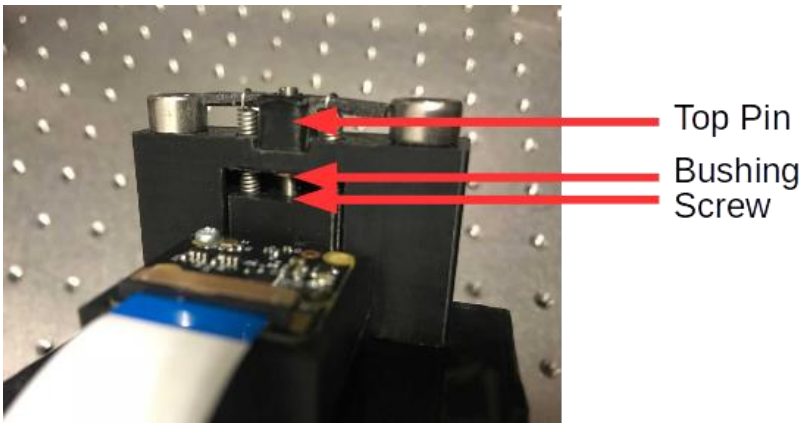

10. Use screws with nuts to fix the focusing stage tightly to the top plate of the sample stage from Supplementary Figure S1 1(b)).

11. Place the focusing stage module onto the sample stage module with the *Raspberry Pi* computer. The part is designed to have a tight fit, if it is not tight, please adjust the tolerances.

12. Optional: connect a screen using the HDMI port.

13. Optional: connect a keyboard and a mouse.

14. Optional: Place the *Raspberry Pi* board on top of the 3D printed base; align the base and the Raspberry Pi such that the screw holes are on top of each other.

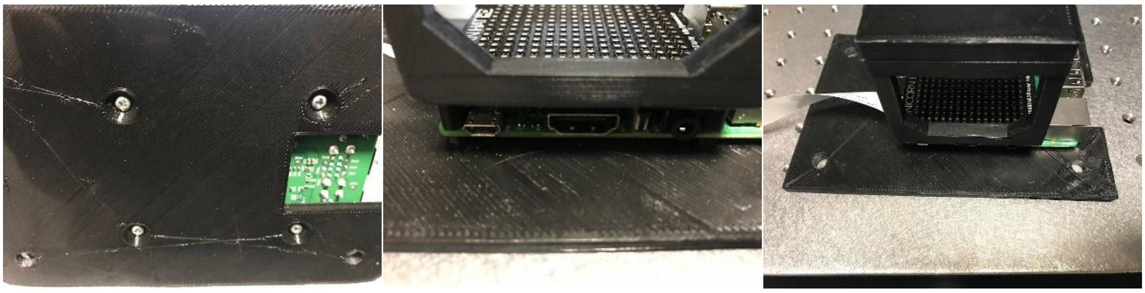

Operating the Microscope

Installing Software on the Raspberry Pi

Raspbian is a free *Raspberry Pi* operating system available for download from the manufacturer's website. It can be installed by following the guide listed in the *Links* section.

The various interfaces of the Raspberry Pi can be enabled by going to Applications Menu -> Preferences -> Raspberry Pi Configuration -> Interfaces, and then enabling all options.

Image acquisition codes can also be downloaded from the *Links* section. Various python packages will need to be installed before these can be used. The packages needed are:

- Unicornhathd
- Numpy
- Picamera
- Matplotlib
- Io
- Random
- Fractions

These can be installed using the pip package management system or by installing anaconda on the raspberry pi. However, the picamera and unicornhathd packages are not included in anaconda and will need to be installed separately. Links to the installation guides of these packages and a more general guide to installing python packages on the Raspberry Pi are provided in the *Links* section. Python2 is used for image acquisition so follow instructions for Python2.7 as opposed to Python3.

We have also provided a cloned Raspberry Pi image which can be cloned onto an SD card: https://drive.google.com/open?id=1Z59lnhNKuGGGVIF1KtoCw2bAcdZESKBo. You can download it and clone onto an SD card by following this tutorial https://beebom.com/how-clone-raspberry-pi-sd-card-windows-linux-macos/. The cloned image contains image capture codes and all the required packages pre-installed for easy plug-and-play operation of the microscope.

Data Acquisition

1. Connect the Raspberry Pi to a keyboard, mouse and monitor, and turn it on.
2. Place the sample on the top plate of the sample stage, underneath the camera mount.

3. Use the "Focusing" script to make sure the sample is positioned correctly and in focus. This script has an option to zoom that can be used if needed. To focus the microscope, use an Allen key to turn the screw in the focusing stage.
4. Close the preview and open the "main data acquisition" file.
5. Adjust the necessary parameters in the data acquisition file and save the file.
6. Place the microscope in a dark room or cover it, being careful to ensure the sample is not moved and the focus is not shifted.
7. Run the data acquisition script.
8. Switch off the Raspberry Pi after data acquisition is complete.

Data transfer

The captured data can be copied by a USB drive or the SD card on the *Pi* can be inserted into a PC and *disk internals Linux reader* can be used to copy the data. Data can also be transferred through the internet or an Ethernet cable. One possible approach is by transferring files using a file transfer protocol (FTP) https://www.dexterindustries.com/howto/how-to-transfer-files-to-your-raspberry-pi-from-a-pc-computer/. Another approach is to transfer files to a cloud storage location (e.g. OneDrive or Dropbox) and retrieve the files with another computer.

Links

- Data acquisition codes and CAD files: http://dx.doi.org/10.5525/gla.researchdata.594
- *Raspbian* installation guide: https://www.raspberrypi.org/documentation/installation/installing-images/README.md
- Needs Etcher software to install Raspbian on the SD Card
- But first, download the Raspbian image from the above link.
- Guide to Installing *Python Packages* on *Raspberry Pi: https://www.raspberrypi.org/documentation/linux/software/python.md*
- *Picamera* installation guide: https://picamera.readthedocs.io/en/release-1.13/
- *UnicornHatHD* installation guide: https://www.github.com/pimoroni/unicorn-hat-hd
- *Pimoroni Unicorn HD* LED array: https://shop.pimoroni.com/products/unicorn-hat-hd
- *Raspberry Pi V2.1* camera: https://www.raspberrypi.org/products/camera-module-v2/
- *DiskInternals Linux Reader* can be used to read the files on a Linux SD card from a computer*:* https://www.diskinternals.com/linux-reader/
- Installing a cloned Raspberry Pi image to an SD card: https://beebom.com/how-clone-raspberry-pi-sd-card-windows-linux-macos/
- Raspberry Pi cloned image used for this microscope: https://drive.google.com/open?id=1Z59lnhNKuGGGVIF1KtoCw2bAcdZESKBo

Supplementary material 2: Reconstructions from the simulations

Simulations were carried out to compare the reconstruction quality from the sparsely sampled Bayer filtered images using (1) standard FPM algorithms on demosaiced images and (2) sparsely sampled FPM reconstruction on raw Bayer data. These reconstruction methods were applied to investigate their performance for various sampling and frequency overlap criteria. Simulations for a non-Bayer image sensor (monochrome) are also presented to provide a reference for the results obtained from a Bayer filtered image sensor (colour). Results are shown in Supplementary Figure S2 1, Supplementary Figure S2 2, Supplementary Figure S2 3. These are the reconstructed images for data points displayed in the graphs presented in Figure 2 of the main manuscript.

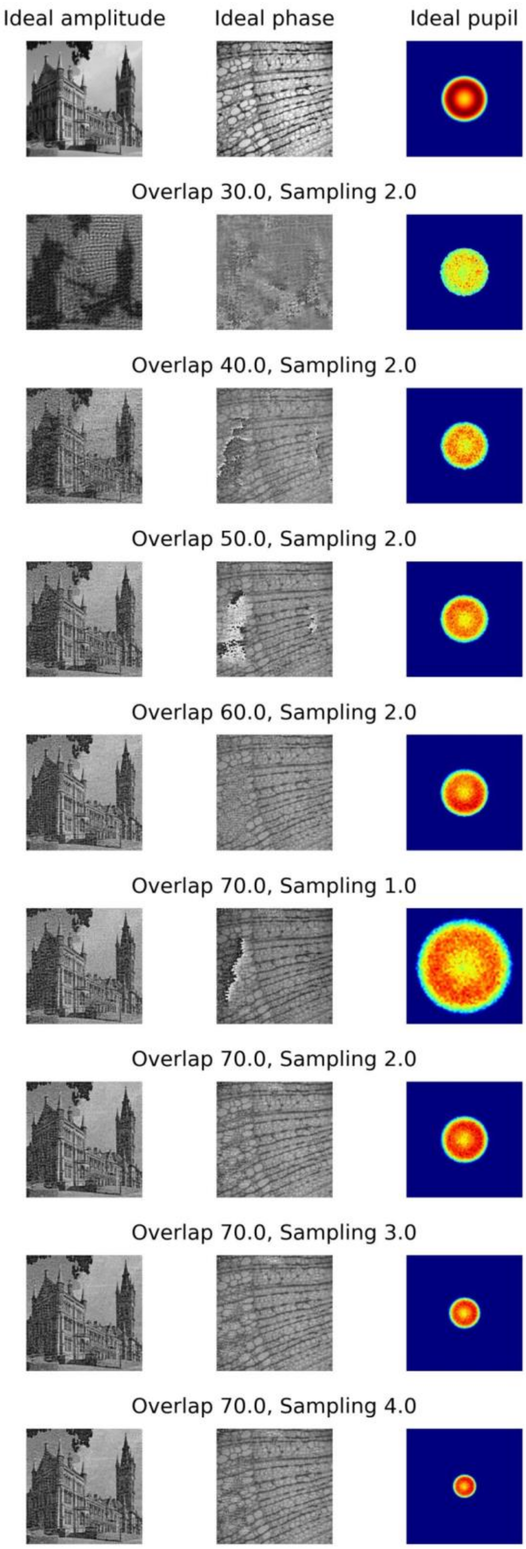

Supplementary Figure S2 1 Reconstructions from images obtained with a monochrome sensor (no Bayer filter) using the standard FPM algorithm. First row shows the expected ideal reconstruction and the remaining rows shows the reconstructions from datasets captured with (1) various image sampling criteria and (2) overlap between the spatial frequencies captured by any two adjacent illumination angles. Noise and aberrations are added in the simulated images to mimic the experimental conditions.

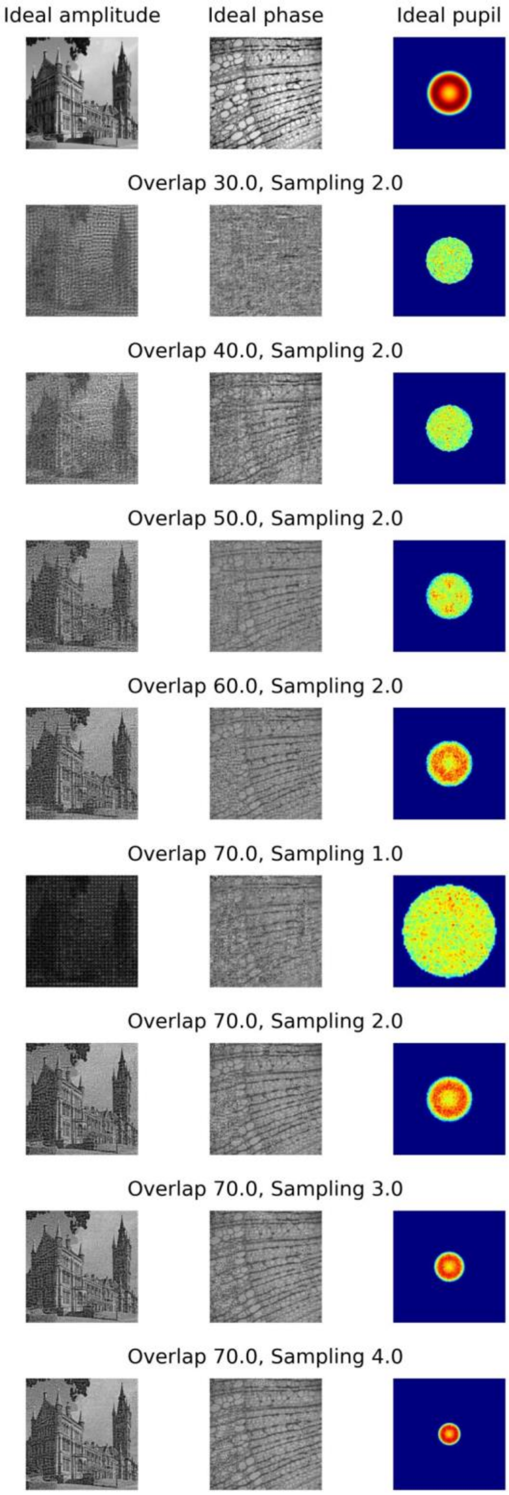

Supplementary Figure S2 2 Reconstructions from images obtained with a color sensor (with Bayer filter array) using the sparsely-sampled FPM algorithm. These images are sparse due to the intermittent sampling from the Bayer filter array. First row shows the expected ideal reconstruction and the remaining rows shows the reconstructions from datasets captured with (1) various image sampling criteria and (2) overlap between the spatial frequencies captured by any two adjacent illumination angles. Noise and aberrations are added in the simulated images to mimic the experimental conditions.

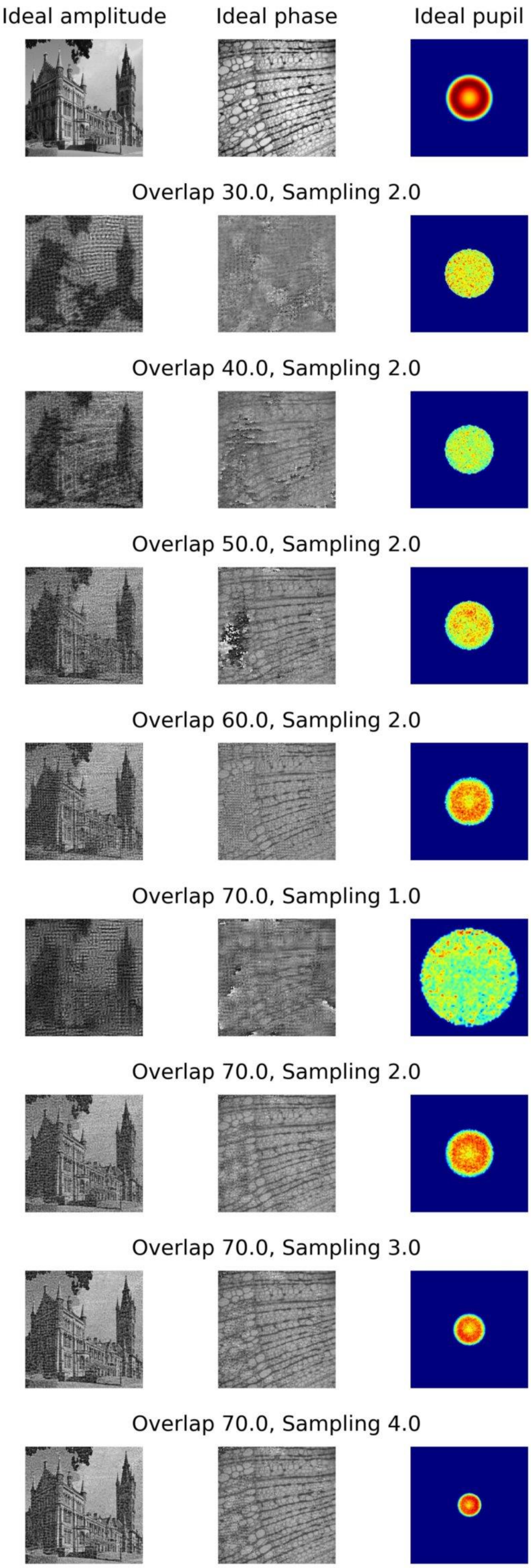
Ideal amplitude
Ideal phase
Ideal pupil
Overlap 30.0, Sampling 2.0
Overlap 40.0, Sampling 2.0
Overlap 50.0, Sampling 2.0
Overlap 60.0, Sampling 2.0
Overlap 70.0, Sampling 1.0
Overlap 70.0, Sampling 2.0
Overlap 70.0, Sampling 3.0
Overlap 70.0, Sampling 4.0

Supplementary Figure S2 3 Reconstructions from images obtained with a color sensor (with Bayer filter array) using the standard FPM algorithm after demosaicing. The sparse images captured from the Bayer filter array are demosaiced (bilinear interpolation) such that the standard FPM algorithm can be implemented. First row shows the expected ideal reconstruction and the remaining rows shows the reconstructions from datasets captured with (1) various image sampling criteria and (2) overlap between the spatial frequencies captured by any two adjacent illumination angles. Noise and aberrations are added in the simulated images to mimic the experimental conditions.
